# Understanding peer support: a qualitative interview study of doctors one year after seeking support

**DOI:** 10.1186/s12913-023-09312-y

**Published:** 2023-04-01

**Authors:** Ingrid Marie Taxt Horne, Frode Veggeland, Fredrik Bååthe, Christina Drewes, Karin Isaksson Rø

**Affiliations:** 1 Research Institute Modum Bad, Postbox 33, Vikersund, 3371 Norway; 2 Institute for Studies of the Medical Profession, Oslo, Norway; 3 Department of Organisation, Leadership and Management, Inland Norway University of Applied Sciences (HINN), Lillehammer, Norway; 4 Health Department, County Governor of Trøndelag, Trondheim, Norway

**Keywords:** Doctor health, Qualitative research, Health services research, Health workforce, Peer support

## Abstract

**Background:**

Doctors’ health is of importance for the quality and development of health care and to doctors themselves. As doctors are hesitant to seek medical treatment, peer support services, with an alleged lower threshold for seeking help, is provided in many countries. Peer support services may be the first place to which doctors turn when they search for support and advice relating to their own health and private or professional well-being. This paper explores how doctors perceive the peer support service and how it can meet their needs.

**Materials and methods:**

Twelve doctors were interviewed a year after attending a peer support service which is accessible to all doctors in Norway. The qualitative, semi-structured interviews took place by on-line video meetings or over the phone (due to the COVID-19 pandemic) during 2020 and were audiotaped. Analysis was data-driven, and systematic text condensation was used as strategy for the qualitative analysis. The empirical material was further interpreted with the use of theories of organizational culture by Edgar Schein.

**Results:**

The doctors sought peer support due to a range of different needs including both occupational and personal challenges. They attended peer support to engage in dialogue with a fellow doctor outside of the workplace, some were in search of a combination of dialogue and mental health care. The doctors wanted peer support to have a different quality from that of a regular doctor/patient appointment. The doctors expressed they needed and got psychological safety and an open conversation in a flexible and informal setting. Some of these qualities are related to the formal structure of the service, whereas others are based on the way the service is practised.

**Conclusions:**

Peer support seems to provide psychological safety through its flexible, informal, and confidential characteristics. The service thus offers doctors in need of support a valued and suitable space that is clearly distinct from a doctor/patient relationship. The doctors’ needs are met to a high extent by the peer-support service, through such conditions that the doctors experience as beneficial.

**Supplementary Information:**

The online version contains supplementary material available at 10.1186/s12913-023-09312-y.

## Background

Ensuring that doctors remain in the workforce is of the utmost importance with respect to sustaining and developing health services as well as with regards to providing the best possible healthcare to patients [1]. In this context, research has found troubling numbers of decreasing satisfaction and increasing burnout among doctors [2, 3]. Doctor burnout is associated with lower quality of care, increased turnover, more frequent medical errors and lower patient satisfaction [4–8]. Doctors’ health and wellbeing are also important for individual doctors [4, 9–12] and their families. To lower the threshold for seeking help, as doctors are hesitant to seek regular treatment [13–16], peer support services are provided in many countries.

*Peer support* is a general description of a concept that initially was used in terms of peers as fellow patients. The NHS defines peer support accordingly: “Peer support is a range of approaches through which people with similar long-term conditions or health experiences support each other to better understand the conditions and aid recovery or self-management” [17]. In the context of this study, peer support is used to describe support from someone with the same professional occupation, but not in the form of a doctor-patient relationship. This type of support can take the form of advice, mentoring, or simply providing a listening ear. In this manner, peer support can lower the threshold for help-seeking as it is a meeting between equals and not treatment. Peer support services may be the first place to which doctors turn when they need help and advice relating to their own health and private or professional well-being [18, 19]. This paper discusses the ways in which a peer support service can meet the needs of doctors who seek help.

Historically, peer support programs date back to the 1970s in the US. police force [20]. In response to reports of poor mental health and increased suicide rates as well as a growing concern for doctors’ wellbeing in the early 1980s, several Physician Health Programs (PHPs) were institutionalized in the US with the aim of preventing malpractice behaviours, mainly in the context of offering treatment of drug and alcohol misuse. PHPs have evolved into effective treatment programs; the success rates found in PHPs significantly exceed those of other addiction care practices [14, 21, 22]. Based on these PHPs, low-threshold collegial services emerged to provide peer support to doctors. Many peer support services are run by doctors on a voluntary basis and practice strict confidentiality. Internationally, peer support services feature different degrees of surveillance and support as well as varying degrees of emphasis on confidentiality [21, 23–25]. Several services do offer confidential support and counselling, although one common limitation is that confidentiality may be broken if a patient’s health is at risk or if a breach is deemed necessary because of serious concerns for the safety, health or welfare of the doctor or another person [26–28]. Norwegian initiatives in this context go far in keeping confidentiality to facilitate for doctors to speak openly.

The peer support service studied in this research is a locally based nationwide peer support network in Norway that is accessible to all doctors and medical students. This network includes approximately ninety dedicated peer supporters across all eleven Norwegian counties. The service markets itself as “an outstretched hand from colleague to colleague”, is free of charge and does not require membership in the Norwegian Medical Association. On the homepage of the Norwegian Medical Association, contact information such as the names, e-mail addresses, phone numbers, towns of residence, and medical specialties of the peer supporters is accessible [29]. The service offers up to three counselling sessions with a peer, and the peer receives monetary compensation from the Norwegian Medical Association. Peer supporters guarantee confidentiality and retain full discretion regarding the information with which they are entrusted. The doctor seeking support freely chooses the peer supporter they reach out to. The service is trust-based and thus no record or identification system is in place. Although the number of conversations with each peer supporter is limited to three, the help-seeking doctor can freely seek another peer supporter for additional support.

The service does not provide medical services, i.e., no medical records are kept, and no sick notes or medical prescriptions are provided. The regular setting of peer support is by a face-to-face meeting. Some doctors seeking support may not want to or are not available for meetings. In these cases, the support may be given by phone or even video link. The peer supporter and the doctor together are free to choose which meeting modality they prefer. The design of the service thereby signals expectations of an informal setting, a safe framework featuring confidentiality, and an open conversation characterized by equality between peers rather than a doctor‒patient relationship. A booklet [30] describing the role of peer supporters is published on the service’s internet page. Here, the peer supporters and help-seeking doctors can find practical advice about the service’s organization and guidelines, for example a description of the confidentiality. For training and quality assurance of the service, the peer supporters must participate at a 2-day, annual national meeting at least every third year. Here they practise giving support by role play and discussion of cases. They also have lectures, case discussions and exercises on the agenda. In some counties, there are additional local meetings for the peer supporters providing an arena for debriefing, supervision and support. The supporters are recruited by appointment of the local medical association in each county. It is considered desirable that a variety of medical specialties are represented among the peer supporters to ensure a broad diversity of collegial needs to be met. Sometimes a peer supporting session is followed by a formal, medical consultation after agreement by the doctor seeking support. This option is also described explicitly in the role description information booklet [30] and is often discussed at the regular, annual meetings of the peer supporters. If this transition takes place, from that point on, the conversation is regarded as a regular medical consultation. Specifically, the peer counsellor changes roles from that of peer supporter to that of medical professional, with all legal and professional duties entailed by the latter.

Long-term studies of PHPs have been conducted [31–33], but only a very limited number of long-term studies have investigated the possible benefits of peer support on doctors. A longitudinal Norwegian study found improved mental health among doctors up to 3 years after attending a peer support service, Villa Sana. That study reported a reduction in doctors’ levels of burnout, job stress, and symptoms of depression and anxiety both 1 and 3 years after their contact with peer support [11]. In a previous qualitative study of the Norwegian Medical Association’s locally based peer support service, the usefulness of peer support for doctors was investigated by interviewing the people providing the service, i.e., the peer supporters [34]. This study concluded that it is necessary to interview individuals who have used the service in order to obtain new knowledge regarding peer support and the ways in which it can meet doctors’ needs. A lack of qualitative studies in the field of doctors’ mental health and wellbeing indicates the need for more explorative and interpretative investigations of this context.

In medical culture, doctors have expectations of being able to cope, to endure, and to avoid suffering from mental illness or showing weakness [35, 36]. This aspect of professional medical culture is often referred to as “the hidden curriculum” [37, 38]. Within such a culture, it can be difficult to feel sufficiently safe to show weakness, insecurity, or illness at work. The design of the Norwegian medical association’s locally based peer support service emphasizes the fact that doctors should be able to speak openly without incurring negative consequences, to ensure a low threshold for doctors who are hesitant to seek help and to offer a venue that is free from the hidden curriculum as well as the traditional doctor‒patient relationship [30]. To better understand how a peer support service can meet the needs of help-seeking doctors in such a context, it can be useful to consider Edgar Schein’s theory and thoughts regarding ways of providing psychological safety and the way norms and culture develop within an organization.

### Theory

#### Conceptions of psychological safety and the development of norms in a social environment according to Schein

*Psychological safety* is an expression originally stemming from Willian Kahn [39] and later described by Amy Edmondson as “people’s perceptions of the consequences of taking interpersonal risks in a particular context such as a workplace” [40, 41]. By utilizing this theoretical lens, we can explore whether the way in which the peer support service studied is designed and practised contributes to “psychological safety”. Additionally, we can gain knowledge about doctors’ thoughts regarding the interpersonal risk they take when they seek support.

In an environment with a lack of psychological safety, the energy, creativity, and potential for development that can be obtained by sharing thoughts is lost. Schein [42] discusses the presence of psychological safety in organizational cultures as necessary for changing and improving a social environment. It is the management’s responsibility to provide a psychologically safe environment, which entails ensuring that subordinates feel sufficiently safe to express their thoughts and opinions. Psychological safety is closely linked to norms of interpersonal communication and sharing of thoughts in professional organizations. Norms function as decisions or rules within a group. Norms can be expressed through formal rules or guidelines (formal norms) as well as cultural expectations (informal norms). On some occasions, these formal and informal norms are in agreement, while on other occasions they are not. According to Schein, an occupational setting includes norms pertaining to a broad range of issues, including how to deal with problems with authority, how to establish workable peer relationships, how much emotion to display, whom to ask for help, and with what issues it is appropriate to ask for help [42]. The hidden curriculum [43] describes certain informal, often unspoken norms that make it difficult for the doctor to expose emotions, weakness, and illness or to admit mistakes in a professional medical setting. The conclusion of a paper by Tait Shanafelt and Schein that discussed several of these elements is that change is necessary to “heal the professional culture of medicine” [44].

Psychological safety is considered to be a basic interpersonal need at the workplace to foster well-being and personal development. Like all basic needs, if the need for psychological safety is not fulfilled in one place (the workplace), the workers will seek to fulfil it somewhere else. This paper studies and discusses the ways in which a peer support service for doctors might contribute to satisfy these needs.

### Aim

We investigated the reasons why doctors sought a peer support service and the ways in which such a service could meet the needs of doctors who seek help.

## Methods

For this study, approximately 450 consent forms for participation were provided to the peer supporters working for the service (90 peer supporters × 5 consent forms each). In accordance with the study design, the peer supporters recruited doctors attending the service with at least one meeting, during the period January–December 2019. The peer supporters chose freely if they distributed the consent forms to the help-seeking doctors. It was the responsibility of the doctors to complete and post the forms. This mode of recruitment was the only one available to us as the peer supporters practise confidentiality and cannot share identifying information. In this manner, the help-seeking doctor chose freely whether to engage in the research project and thus to share sensitive information. Of the 450 consent forms distributed to the peer supporters, 22 consents were returned. When we started recruitment in January 2020, several doctors had difficulties to take time out of their busy schedules to meet face-to-face and thus withdrew from the study. After the Covid -19 pandemic arrived recruiting for on-line interviews was easier. This resulted in 12 interviews, see Table 1.

**Table 1:** Participants

Characteristics of the selected participants (*n* = 12)	Participants (n)
**Gender**	
Male	3
Female	9
**Age** (average 46 years)	
60–75	3
50–60	2
40–50	2
30–40	4
20–30	1
**Medical specialty**	
Family medicine	5
Surgical specialties (gynecology, otolaryngology)	1
Laboratory medicine (biochemistry, radiology)	2
Psychiatry	1
Internal medicine (geriatrics, internal and occupational medicine)	3
**Work experience**	
0–10 years	4
10–20 years	5
20–30 years	1
30 + years	2

The interviews were conducted a year after each individual’s attendance to peer support (± 20 days), as planned, during 2020. The mode of recruitment used in this study represents a criterion-based case selection, the only criterion being attendance at the local peer support service in 2019 [45]. The first and last authors, both females, conducted semi-structured interviews lasting between 50 and 90 min. Ten interviews were conducted on-line with video and two interviews were conducted by phone according to the interviewees wishes. Interview data were complemented by supportive notes to aid discussion of perspectives and interpretations after each interview. The interviews were audiotaped, not videotaped, and transcribed by a trained research assistant. In the study design, the interviews were planned as face-to-face meetings but had to be re-scheduled as mentioned above, due to national rules during the COVID-19 pandemic in 2020 rendering physical meetings impossible.

Qualitative research methods facilitate the investigation of experiences, beliefs, and values. The systematic collection, organization, and interpretation of interviews make it possible to explore the meanings of social phenomena as they are experienced by individuals themselves.

After reading through the empirical material, the preliminary themes were identified and discussed between all authors. The texts were analysed using NVIVO software with systematic text condensation [46], as described in Table 2, by the first and last authors in cooperation. This produced generalized descriptions in thematic code groups reflecting among other things the characteristics sought by doctors from the peer support service. Concerning reflexivity, the interviewers are both doctors (with specialty in occupational medicine and in psychiatry) and thus shared tacit knowledge with the interviewees regarding medical culture and professionality must be assumed. This is a strength as it facilitates understanding the interviewees situation but can lead to lack of questioning of cultural phenomena taken for granted within the profession. Both interviewers work as counsellors for colleagues (in another service) and are thus also trained in non-therapeutic dialogue, as well as having conducted qualitative interviews in the past. The first author is a PhD candidate, the last author is a senior researcher, PhD. The participants received oral and written information about the project and the interviewers. The co-authors are the user representative (also a doctor), a professor in political science teaching research design and health policies and a post-doc in medical sciences with research areas including doctors’ professional identity and professional fulfilment. The descriptions and concepts were discussed among all five authors until consensus was reached. Finally, the first author applied these concepts to an analysis of all the interviews. This analysis was data-driven, although it was supported by theories of organizational culture to improve our understanding further. The results section was sent to the interviewees, who approved the text and the citations and did not suggest changes. Table 2 demonstrates stepwise the analytical approach.

**Table 2:** Procedures for analysis according to Systematic Text Condensation [46, 47]

1. Total impression – from chaos to themes	Reading through the material to get a general impression of the whole. Looking for preliminary themes associated with the research question
2. Identifying and sorting meaning units – from themes to codes	Identifying meaning units, give them a code. A code is a label gathering connected meaning units into groups
3. Condensation – from code to meaning	The thematic code groups across individual participants constitute an analytical unit for further abstraction by condensation of content
4. Synthesizing – from condensation to descriptions and concepts	From the condensates, we develop credible narratives that can make a difference by providing descriptions and concepts that inform the study question

### Patient and public involvement

Patients were not involved in this study. A user representative (a doctor who had both sought peer support and served as a peer supporter) was involved in planning the study, writing the project description, analysing the data, and co-authoring the present paper.

## Results

Although the doctors sought peer support for a variety of reasons, they all had difficulties managing their work situation in combination with private stressors. They expected support on an equal basis from a peer who could understand their situation. It was important that the peer supporter and the setting was distinct from a doctor-patient relationship and offered a psychologically safe space for the conversation. Below, we elaborate these findings in three main categories.

### Reasons for attending peer support

The doctors sought peer support for a variety of reasons, but they all mentioned difficulties coping with their work situations and the desire to seek a “free space” to talk outside of the workplace. They discussed signs of burnout, such as fatigue, reduced work capacity and a feeling of being more vulnerable than before.

*…I thought I had to get help to think differently in relation to my everyday life because I had reduced work capacity and felt much more vulnerable…*

*Interview11*

The doctors sought advice concerning career changes, retirement, and illness. Some experienced a lack of support from and companionship with their colleagues. Others described bearing excessive responsibility, encountering anxiety linked to work, or feeling inadequate at work. They discussed ways of dealing with death threats from patients and challenges associated with taking up a new position or sought support after experiencing a personal crisis such as divorce. Some regretted having chosen the medical profession and asked for advice regarding ways of coping with work and a profession that they did not enjoy.

*No, I guess that I didn’t quite know how to handle being a doctor when I didn’t have any desire to be one, and I also wanted to get some motivation to at least finish the internship.*

*Interview 6*

Some doctors sought help with symptoms of depression, trauma or anxiety and deliberately reached out to a peer psychiatrist.

### Hopes and expectations

#### Seeking support and understanding from a fellow doctor

The doctors used the service to receive support from a peer on an equal basis and to discuss ways of handling stressful situations at work or chaotic life situations. It was important that the peer supporter understood what it means to be a doctor.

*I felt that the situation was sort of chaotic, and I probably hoped to get some help to sort through it and to get […] thoughts from other doctors about how they would proceed in such a situation, for example, what would they have let go and what would they have focused on.*

*Interview 3*

Doctors mentioned their experiences of a lack of sufficient support or feeling uncomfortable discussing their concerns at work. They noted that they did not know where else to turn to discuss work-related issues. They appreciated receiving feedback from a doctor who was not involved in their situation and who did not have a close connection to their place of work. Some doctors sought motivation and advice regarding how they could handle being a doctor despite their feelings of regret regarding their choice of profession. Several attended the service to deepen their understanding of their experiences of reduced work capacity and fatigue. A few sought specific advice regarding ways of solving different issues at the office and contacted a peer supporter who worked in the same medical specialty.

*We discussed a lot of specific issues concerning practicalities, about the health secretary and how I could proceed there and what experiences she [the peer supporter] had. And yes, I got a bit of support […] that it is tough to have leadership responsibility for employees as a GP. And that it is completely different than medical issues. So, I got some ideas about things that I am not very good at and with which I have no experience. That was a good conversation.*

*Interview 12*

Some doctors specifically chose a peer who was a trained psychiatrist, either because they considered their issues to be primarily related to mental health or because they thought that a psychiatrist would have specific competence relevant to the issue at hand, for example, the receipt of death threats from patients at work and ways of handling that issue. Finally, peer support was used as a venue to discuss situations that were challenging in the context of both work and private issues.

*I don’t think it’s okay […] to talk about what is problematic at work. There aren’t many I could do that with, I think, so that was probably part of the reason [that I sought peer support]. And I was exhausted and wondered what I could relate to work and what it was with me that just wasn’t functioning.*

*Interview 7*

#### Seeking psychiatric expertise

Doctors who deliberately chose to seek a psychiatrist as a peer supporter also sought advice regarding ways of coping with excessive workloads and challenges at work. Additionally, these doctors more or less expressed their intention to seek psychiatric treatment, despite the fact that treatment is not offered by the peer support service.

*...but when I contacted her [the peer supporter], it was […] both as a therapist and due to the fact that I didn’t quite know what was happening to me, but it was in connection to work, and I thought it would be good to have chat with her and that she might have some input.*

*Interview 1*

*... I probably started to get depressed as well as very, very exhausted. I cried a lot, didn’t remember the PIN to my credit card, went into the mall and didn’t know where to exit. So, I think I just needed a person to discuss it with, who could give me advice, ideas on how to get out of it…*

*… I thought I needed a psychiatrist.*

*Interview 12*

Some of the doctors were offered formal treatment and noted the existence of partially unclear boundaries between peer support and health care. Despite such unclear boundaries, the help-seeking doctors reported that they were cared for very well. In situations in which doctors hoped for treatment, they could experience disappointment if the peer supporter explicitly focused on a merely supportive role.

### A relational space

#### Valued traits of peer supporters

The doctors appreciated meeting with a peer supporter who responded quickly and who was sufficiently flexible to meet outside of regular working hours. The opportunity to influence the decision regarding where and when to meet was highly appreciated by the help-seeking doctors. The peer supporters were perceived as trustworthy, which enabled openness in the context of difficult topics. Being met with courtesy and respect and being given the time necessary to unburden themselves were described as key assets by a peer supporter.

*For me, it was decisive that he (the peer supporter) recognizes my problem and sets aside time.*

*Interview 9*

Some doctors valued the encouragement to speak freely, while others appreciated being asked interested questions by the peer supporter. Some reached out to a peer supporter who worked in a specific medical specialty for professional advice. Several experienced the conversation(s) with the peer supporter as an opportunity to find a way of coping with the situations they faced.

*She invited me to come back, and she explained the framework for the service and all that. I just have to say, I’m very appreciative and very satisfied with all that. I felt a bit like, after a couple of conversations, it was as if I got back on my feet again […] and that was very good.*

*Interview 7*

#### Setting

The doctors wanted to be offered a setting that was distinct from the settings that typically characterize doctor/patient interaction. The meetings took place at the peer supporter’s home, in the peer supporter’s office or in a break room after hours.

*Yes, I turned up at her office, I guess it was actually after work hours, and she offered to meet at her office or outside or in a café […] That was also very nice because then it felt possible to choose something that felt okay to me.*

*Interview 7*

One of the interviewees was only offered the opportunity to meet during regular working hours, which limited the number of meetings available to the help-seeking doctor due to conflicting work obligations. Several interviewees were offered something to eat or drink. It was emphasized that the setting should be safe and caring but should also feature clear boundaries, thus ensuring a balance between a professional and a private setting.

*We had the conversations in her break room after working hours; it was peaceful and quiet there. It was nice not to be in an office, but we were in a break room, and she had some fruit and coffee, and the setting was very good.*

*Interview 11*

One doctor chose to receive peer support by phone. One doctor was met with the same physical structures as those associated with a doctor/patient relationship; that is, the meeting was conducted at the office and the help-seeking doctor was not offered anything to drink, which caused the doctor to feel that inadequate care was provided.

*…it would have been nice to have something that was a little more friendly and open and maybe a, I don’t know, a cup of coffee or something that you can be offered when you get a haircut, for example.*

*Interview 8*

#### Formal structure of the service

The doctors appreciated the fact that the peer supporter gave them a brief introduction to what the service could and could not offer. It was described as a relief to be able to step out of their regular roles as doctors or patients and to be given the opportunity to speak freely without the constraints that accompany a medical consultation.

*…maybe it was less stigmatizing when there wasn’t a medical record.*

*Interview 3*

The confidentiality of the service was highly valued. The doctors noted that such confidentiality provided them with a sheltered space in which they could discuss work-related problems and difficulties involving their colleagues, since workplace culture could make it challenging or impossible for them to discuss these topics at work.

*… I need someone to simply help me sort through my thoughts […] when things are kind of difficult at work, I almost don’t know who to turn to.*

*Interview 7*

In addition, the service was used by doctors who did not consider themselves to be ill or to have health-related issues that naturally belonged in a patient record.

*If I had been ill, it would have been correct to keep a medical record, but just being human doesn’t need to be recorded, I think.*

*Interview 6*

Some doctors found it to be less stigmatizing to seek a type of support that did not involve any record-keeping.

*It’s a bit about being removed from the role of doctor and the role of patient and being allowed to talk about what is important to you. As soon as a medical record is mentioned out loud, it takes on a professional touch, which can perhaps defeat the purpose to some degree.*

*Interview 4*

*I didn’t have the feeling that it (the issue at hand) was something that should be diagnosed, that it was more of a life crisis or what I should call it, something that everyone can experience without it being an illness.*

*Interview 3*

## Discussion

The main result of this study was that the doctors wanted peer support to be qualitatively different from that of a regular doctor/patient appointment. They sought support due to a range of different needs and discussed occupational, personal, and private challenges. They attended peer support either to engage in dialogue with a fellow doctor or in search of a combination of dialogue and mental health care by seeking peer supporters with psychiatric expertise. The doctors valued flexibility concerning where and when to meet, the lack of a medical record, a confidential setting, an informal setting that differed from the patient‒doctor relationship, and the availability of sufficient time to allow them to speak freely. Some of these factors are related to the formal structure of the service (such as its confidentiality), whereas other elements are based on the way in which the service is practised and on regular discussions of, and consensus regarding this practice in formalized annual meetings among peer supporters.

### Reasons to attend peer support

The broad range of reasons why doctors attend a peer support service reported by this research all include involvement of issues at work as well as private stressors. This is in line with findings of previous research, which consistently report work-related difficulties to be relevant in this context [18, 19, 48]. Literature shows that the structure of the peer support offered, whether it is designed as peer support [18, 19] or as a coaching intervention [48], also influences the type of issues that are brought up by the services’ users. Additionally, in this study, we found that peer support was used to meet a combination of individual needs and needs related to work issues.

### Hopes and expectations

The empirical material demonstrates that interviewees’ hopes and expectations were met to a large extent. The doctors searched for and received a friendly, inquiring, and attentive doctor colleague as well as an informal place to reflect. This description is in line with the motto of the peer support service: “An outstretched hand from colleague to colleague”. The findings resonate with previous research where acknowledgment, reflective listening, and support are found to be essential qualities of peer support [49, 50]. The doctors sought dialogue and, to a lesser extent, specific input regarding ways of solving a problem. Through peer support, the participants were offered confirmation of their perspectives and experiences as fellow doctors. The empirical material indicates that the doctors wanted and received personal and professional recognition from the service. The sense of this personal and professional recognition was said to be due to the formal structure of the service, the context in which the service took place, and the traits of the peer supporters. This is in agreement with previous research which finds recognition to be an important condition to support physician engagement [51, 52].

Doctors’ reluctance to seek formal health care, especially with regard to mental health difficulties, has been well documented [16, 53–57]. This is further confirmed in this study by the fact that doctors seek psychiatric expertise in the context of a peer support service that explicitly does not offer medical treatment. Based on the empirical material, we know that several peer supporters changed the context of the meeting(s), from offering peer support to offering a formal medical consultation. Thus, by attending peer support, some participants in our study received the treatment they actually needed. These doctors underwent treatment as a continuation of peer support, bridging the gap between collegial conversation and formal treatment. They described the combination between supportive and therapeutic roles at the service. Although this mixing of roles was characterized as unclear, they were highly appreciative of the support and treatment they received. This practice may offer both advantages and disadvantages; on the one hand, it can help ensure that the doctors’ needs are met, while on the other hand, it may weaken a factor that many doctors experience as valuable, namely, the clear distinction between support and the doctor/patient relationship. A study of another Norwegian peer support service that did not offer the possibility of referring the receiver for a medical consultation, found that some help-seeking doctors had hoped for such a possibility [18]. To some doctors, peer support was the first situation in which they felt sufficiently safe to voice their mental health concerns to a colleague, and they would have greatly appreciated a seamless transition into therapy [18]. Taking this into account, the fact that the Norwegian Medical Association’s’ locally based peer support service offers a flexible service that can accommodate these different needs seems to be appropriate.

### A relational space with psychological safety

It was clear in the interviews that the doctors lacked psychological safety at their place of work and sought an open conversation in a flexible and informal setting when attending peer support. Peer support offered by the Norwegian Medical Association has become a well-known institution to which doctors can turn for help [58, 59]. The service provides easy access to help with both work-related and personal issues. The fact that the Norwegian Medical Association owns and manages peer support services might reinforce and legitimize help-seeking. Peer support can function as a haven where doctors can discuss the problems resulting from conditions in the workplace. Part of the reason doctors benefit from this offer is the confidentiality and the provision of a psychologically safe environment.

Some researchers argue that psychological safety of employees, such as valued feed-back and openly admitting to mistakes, is not part of the organizational tradition in the field of health care [44, 60]. To create psychological safety, Schein suggests several activities that can be implemented including a focused dialogue with the goal of helping participants to relax sufficiently to examine their own assumptions and to be able to consider other assumptions as equally valid or true. The accounts of the manner in which the doctors were met at the peer support service are in accordance with a setting which fosters psychological safety as described by Schein [42]. Although peer support does not represent an identical setting to that of Schein’s focused dialogue, it contains many of the same elements which are necessary to encourage reflection. Namely provision of resources and a safe space to discuss difficulties with someone with a similar background [42]. All the doctors included in this study noted that they felt sufficiently safe to discuss their challenges when they attended peer support. The empirical material demonstrates the relevance of both personal characteristics (the peer supporter’s approach to the problem) and structural features (the setting in which the conversations took place, the confidentiality of the service) to the experience of a safe framework and the opportunity to speak freely and openly. Previous studies called for the need to question professional norms and the underlying and often unspoken professional assumptions, the hidden curricula, that can hinder the doctor’s ability to provide self-care [35, 36]. Peer support can thus help the doctor to initiate changes in his or her work and private life or seek adequate treatment. Simultaneously, confidentiality (and thus the associated safe framework) may prevent doctors from addressing problems and provide feedback at their workplace. It could be argued that a factor that allows individuals to benefit from the offer can contribute to limiting efforts to speak up and solve issues at the workplace.

### Implications

Several topics emphasized in peer support conversations are known drivers of burnout among doctors: lack of support, fear of voicing concerns at work, excessive workloads, work–home conflicts, negative leadership culture and a lack of comfort with their amount of responsibility at work [3, 4, 8, 44, 61–66]. In the interviews, explicit statements were made indicating that speaking up at work entails taking a personal risk and that some of the interviewees did not know where else to turn for help with their work-related problems. This finding is in line with the conclusions of recent research suggesting that health care leadership must discover ways of increasing voice and decreasing silence among health care professionals [60]. Such an approach is likely to improve health workers physical and mental well-being.

In his theories, Schein notes that issues might arise with regard to commonly accepted norms pertaining to how to relate to each other, how to deal with problems with authority, how much of one’s personal life to share, whom to ask for help and with what issues it is appropriate to ask for help [42]. To ensure psychological safety at a workplace, Schein suggests the implementation of support groups in which difficulties can be discussed with peers as well as the provision of resources, i.e., the allocation of time and space, necessary to facilitate coaching and the valid feedback that is required to create a psychologically safe space. Accordingly, the lack of a psychologically safe work environment should not be addressed exclusively outside the workplace. Individual doctors may manage to cope with the situation for some time by optimizing their own stress tolerance. Nevertheless, to support doctor well-being and prevent doctor burnout, it is important to ensure that the discussion concerning work issues and psychological safety also happens in the workplace, which is the venue in which many of the reported problems arise. For some issues, it could be considered whether the workplace needs change more than the doctors. It is the health care organizations’ responsibility to provide workplace venues with sufficient psychological safety to address these important challenges and find strategies to create a sustainable work environment. Burnout, fatigue [8, 10, 61, 62, 67, 68] and decreased job satisfaction [4, 69–72] represent problems faced by doctors throughout Europe and the US, and must be addressed at both the individual and organizational levels [44].

This study adds further evidence to the knowledge that doctors are hesitant to seek help and that some of those attending peer support need healthcare. Further, the study narrates aspects important to help-seeking doctors to feel well taken care of when seeking help. Among those aspects are both the physical setting and the attitude and approach of the peer supporter. We think that this information may be of value to employers in healthcare, doctors’ associations, the community of doctors and organisations providing peer support services for doctors internationally.

### Strength and weaknesses/ethics

The digital interviews conducted made the task of information gathering challenging, as the interviews required participants to trust researchers sufficiently to disclose sensitive personal information. Such trust can be more difficult to obtain in the context of an on-line interview. On the other hand, when we started recruitment in January 2020, the doctors were reluctant to take time out of their busy schedules to meet face-to-face. In this period several of the 22 received consents withdrew from the study. After the Covid -19 pandemic arrived, in our experience, recruiting interviewees was easier when the appointment was on-line instead of face-to-face. Maybe as no one was required to travel to a meeting and the digital interview took less time from a busy workday. It is also possible that it was perceived as less invasive to meet digitally.

The sampling process may have led to selection bias. Both peer supporters (who delivered the consent forms) and individuals who sought support (potential participants) may have been sceptical regarding the study. It is possible that the deeply personal nature of the topics discussed in the peer support service led to lower response rates [73]. This is a weakness of the study which can reduce the internal validity. However, in a qualitative study, we seek richness and depth of data that in this case describes a wide spectre of reasons to seek peer support and gives indications of how it is useful to offer peer support to doctors. The internal validity was further strengthened by involving a user representative who also has experiences from being a peer supporter, and by participants approving the quotes used. The relevance of the findings in this study is strengthened by similar findings in other studies [18, 19, 34]. This strengthens the generalizability of the results. Another weakness could be that some interviewees also received regular medical therapy at the peer supporters’ office in continuation of the peer support. This may have influenced their recollection of the support provided.

The results presented are based on the condensates that include information across all the interviews pertaining to the relevant analytical unit. Additionally, we have used illustrative quotes to give examples. Although a few of the interviewees are not directly quoted, their data are thus still represented in the text. The direct quotes have been chosen as they are representative for the overarching findings of the data set. We chose quotes with an emphasis on theoretical generalization; quotes that illustrate the relevance of the theory used (psychological safety) as described in the literature.

Concerning reflexivity, the two interviewing authors are doctors and peer counsellors (although they do not work in the Norwegian Medical Association’s locally based peer support service, but in a centralized service presented in a previous paper [18]). They are also members of the medical professional culture studied. This identity was important for making the participants feel at ease, but it may have led to bias since the authors were investigating their own profession [45]. To balance this bias, the author group additionally consisted of a user representative (a doctor and peer supporter) as well as two authors with different academic backgrounds, but extensive knowledge of the fields of medical professionalism, leadership and organizational change as well as qualitative research. The presence of a co-author who also is a user representative having attended peer support and who has worked as a peer supporter is a strength of this paper. This validated both participant experiences and provided expert knowledge on how the peer support service is managed in practice. The interdisciplinary analysis group has attempted to provide a rich and nuanced understanding of the empirical material [45]. The inescapable influences of researchers have been integrated into discussions concerning the recognition and interpretation of relevant topics, including the task of investigating one’s own professional culture [74].

## Conclusions

The peer support service studied in this research is aimed at help-seeking doctors with a variety of needs and provides a venue for discussing the challenges that result from both individual and work-related factors. These needs are met to a large extent by the peer support service, through conditions that the doctors experience as beneficial. Peer support can provide psychological safety by offering a flexible, informal, and confidential service that is clearly distinct from a doctor/patient relationship. Continuing the offer of a peer support service thus seems important although for some issues the need of change applies as much to the medical workplaces as to the doctors.

## Supplementary Information

**Additional file 1.** Interview guide - Reasoning about contacting peer support in 2019, interviews carried out 1 year later, in 2020.

## Data Availability

All data generated and analysed during this study are stored securely, offline at Modum Bad, Norway. The datasets generated and analysed during the current study are not publicly available due to the personal character of the interviews but are available from the corresponding author on reasonable request.

## Abbreviation

PHPs: Physician health programs
