# Supplementary material for: Understanding peer support: a qualitative interview study of doctors one year after seeking support
Source: BMC Health Serv Res. 2023 Apr 1;23:324. doi: 10.1186/s12913-023-09312-y (PMC10066008; doi:10.1186/s12913-023-09312-y)
Supplement: Supplementary file 1 — Additional file 1. Interview guide - Reasoning about contacting peer support in 2019, interviews carried out 1 year later, in 2020. [file 12913_2023_9312_MOESM1_ESM.docx]

*Interview guide - Reasoning about contacting peer support in 2019, interviews carried out one year later, in 2020*

Intro:

***Can you tell us a bit about yourself and your situation (age, work experience, marital status, your health, children…)***

Questions:

***Individual level***

*- Where did you first hear about the peer support service?*

*- Why did you choose to seek out a peer supporter and not another service (GP etc.)?*

*- Was there something in deciding to contact a peer support that was redeeming in itself?*

*- Why did you contact this particular peer?*

- *Would it be okay to be referred to another peer supporter who has less workload?*
- *How important is geographical proximity to the peer? Sex?*

*- What situation made you seek advice from a peer?*

- *Can you tell us about the main areas that were/are particularly challenging in your situation?*

*- What did you want to get out of the counseling?*

*- What was it about the counseling that was meaningful to you?*

*- How would you describe your situation now?*

- *Which changes are important in your situation now compared to before contact with the peer support service?*
- *What steps have you taken in your life after the contact?*
- *Have you made practical changes in your work or personal situation?*
- *Do you now think differently about your work or personal situation?*

*-Have you visited the healthcare system as a result of the contact? (Sick leave?)*

*-What coping strategies have you used? (Physical activity, ways of thinking, alcohol drugs…)*

***Institutional level***

- *Is it important that the service maintains confidentiality?*
- *Has the contact with the peer support service made a difference for you?*
  - *What has been of importance?*
  - *Which role does the peer support service play in that (possible) importance?*
  - *Which role do other factors play/have?*
- *Which experiences have been useful for you from the contact with the peer support?*
- *What was most important for you in your encounter with peer support?*
- *What were strengths and weaknesses in the contact with peer support?*
- *Is there anything that could have been better?*
  - *What can/should be changed?*

-Are there any questions you had waited to be asked about/wished to be asked about here today that we haven't posed to you?
